# Global *Wolbachia* prevalence, titer fluctuations and their potential of causing cytoplasmic incompatibilities in tsetse flies and hybrids of *Glossina morsitans* subgroup species

**DOI:** 10.1016/j.jip.2012.03.024

**Published:** 2013-03

**Authors:** Daniela I. Schneider, Kathrin I. Garschall, Andrew G. Parker, Adly M.M. Abd-Alla, Wolfgang J. Miller

**Affiliations:** a Laboratories of Genome Dynamics, Department Cell and Developmental Biology, Center of Anatomy and Cell Biology, Medical University of Vienna, Vienna, Austria; b Insect Pest Control Laboratory, Joint FAO/IAEA Division of Nuclear Techniques in Food and Agriculture, Vienna, Austria

**Keywords:** *Glossina*, *Wolbachia*, Symbiont diversity, Inter-species hybrids, Speciation

## Abstract

We demonstrate the high applicability of a novel VNTR-based (Variable-Number-Tandem-Repeat) molecular screening tool for fingerprinting *Wolbachia*-infections in tsetse flies. The VNTR-141 locus provides reliable and concise differentiation between *Wolbachia* strains deriving from *Glossina morsitans morsitans*, *Glossina morsitans centralis*, and *Glossina brevipalpis.* Moreover, we show that certain *Wolbachia-*infections in *Glossina* spp. are capable of escaping standard PCR screening methods by ‘hiding’ as low-titer infections below the detection threshold. By applying a highly sensitive PCR-blot technique to our *Glossina* specimen, we were able to enhance the symbiont detection limit substantially and, consequently, trace unequivocally *Wolbachia*-infections at high prevalence in laboratory-reared *G. swynnertoni* individuals. To our knowledge, *Wolbachia*-persistence was reported exclusively for field-collected samples, and at low prevalence only. Finally, we highlight the substantially higher *Wolbachia* titer levels found in hybrid *Glossina* compared to non-hybrid hosts and the possible impact of these titers on hybrid host fitness that potentially trigger incipient speciation in tsetse flies.

## Graphical abstract

**Graphical abstract:**

**Figure:**
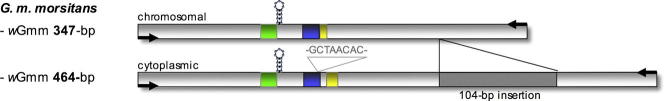

**Highlights:**

► VNTRs are highly diagnostic tools for fingerprinting *Wolbachia* in tsetse flies. ► Multiple infections, free and nuclear insertions into host chromosomes, do exist. ► Some infections can escape detection *via* hiding as low-titer infections. ► In hybrids *Wolbachia* can transform into pathogens by loss of replication control.

## 1 Introduction

*Wolbachia* are universal endosymbionts of most terrestrial arthropods and filarial nematodes that are mainly transmitted from mother to progeny and affect host biology in many ways. For example, these α-proteobacteria are capable of triggering a diverse repertoire of life-history traits in insects such as cytoplasmic incompatibility (CI), sex ratio distortion, longevity, innate immunity, locomotion, olfaction, toxin-sensitivity as well as sexual mating behavior changes (recently reviewed in Schneider et al. (2010)). *Wolbachia* are, next to the γ-proteobacteria *Sodalis glossinidius* and *Wigglesworthia glossinida*, part of the triple-symbiont association present in tsetse flies (reviewed in Aksoy and Rio (2005)). Over the last years, numerous studies have focused on the complex interactions exhibited between tsetse flies and their symbionts (Aksoy and Rio, 2005). This extensive research is of great value and interest not solely for the symbiosis research community, but also for medicine-related fields as tsetse are key vectors of disease. Within the genus *Trypanosoma* (*Kinetoplastida*), particularly the species *T. brucei*, *T. congolense* and *T. vivax* are important disease causation parasites. The sub-species *T. brucei rhodesiense* (*Tbr*) and *T. brucei gambiense* (*Tbg*) are the causative agents of Human African Trypanosomiasis (HAT or sleeping sickness). *T. b. brucei* (*Tbb*), *T. congolense* (*Tg*) and *T. vivax* (*Tv*) cause Animal African Trypanosomiasis (AAT or Nagana), primarily in cattle. Of the 32 tsetse fly species all tested species have been shown experimentally to be able to transmit trypanosomes, but only nine of these, belonging to the *Glossina palpalis* and *Glossina morsitans* groups, normally transmit sleeping sickness. Members of the *palpalis* group (*G. palpalis*, *Glossina fuscipes*, and *Glossina tachinoides*) are the main vectors for *Tbg*, whereas *Tbr* is mostly transmitted by species of the *morsitans* group (*G. morsitans*, *Glossina pallidipe*s; www.who.int). According to the World Health Organization (WHO), 23 out of 25 sub-Saharan countries in Africa were reported HAT-infested between 2000 and 2009 (Simarro et al., 2010). Currently, no vaccines against sleeping sickness are available and treatment options are generally very limited. Thus, novel strategies for fighting this health burden are urgently required. So-called biological pest control strategies, targeting the vector biology, have become highly attractive. One such strategy is the classic sterile insect technique (SIT) that relies on eradication of insect populations by releasing irradiation-generated sterile males into the field (Knipling, 1955). Lately, alternatives to the aforementioned method based on targeting host–symbiont interrelation, are being investigated.

*Wolbachia*-infections in insects were shown to cause reproductive phenotypes such as cytoplasmic incompatibility CI, functioning as a post-mating barrier to hybrid formation (recently reviewed in Saridaki and Bourtzis (2010)). CI results in high levels of embryonic lethality among the offspring and can be either uni- or bidirectional. Unidirectional CI occurs when *Wolbachia*-uninfected females mate with infected males; bidirectional CI arises in mates where both partners harbor different, incompatible *Wolbachia*-infections (recently reviewed in Merçot and Poinsot (2009)). Hence, *Wolbachia*-induced CI could be exploited to induce natural reproductive sterility in tsetse fly populations and consequently hinder the transmission of *Trypanosoma* (Sinkins and Gould, 2006, Rasgon, 2008, Brelsfoard and Dobson, 2009). This idea, which was already discussed in the 1940s (Potts, 1944, Vanderplank, 1944), is currently revived, particularly with respect to a very recent study. Alam et al. (2011) have reported on the expression of strong unidirectional CI in crosses of *Wolbachia*-infected *Glossina morsitans morsitans* to antibiotic-treated ones, pinpointing the biological significance of these symbionts in tsetse flies (Alam et al., 2011). These recent data suggest that tsetse fly *Wolbachia* can cause CI, perhaps not only in *G. morsitans morsitans* hosts but also in other *Glossina* species.

During the past decades, hybridization experiments have been conducted between various members of the genus *Glossina*, producing female hybrids with reduced fecundity and sterile male hybrids under laboratory conditions (reviewed in Gooding (1990)). Moreover, natural hybridization events between *Glossina* species in the field have been reported repeatedly, suggesting that pre-mating barriers to hybrid formation are rather weak between sympatric members of this genus (Corson, 1932, Potts, 1944, Vanderplank, 1944, Vanderplank, 1947, Vanderplank, 1948, Gooding, 1993). Extensive studies on mating behavior have uncovered that *Glossina swynnertoni* females, for example, are not very choosy and accept all mates regardless of whether they are con-specific or not (Gooding, 1993). *G. swynnertoni* males attempt to mate equally with females of *G. swynnertoni*, *G. m. morsitans* and *G. m. centralis*, regardless of con-specificity of the mates (Gooding, 1993) and *G. swynnertoni* males are able to both inseminate and fertilize *G. m. morsitans* females (Gooding, 1993, Gooding, 1997). Hence in the evolutionary point of view the *G. morsitans* group is considered a very young and highly dynamic species complex with weak pre-mating isolation but significant post-mating barriers by expressing high hybrid mortality and complete hybrid male sterility (Gooding, 1999).

Since unidirectional incompatibilities were observed in crosses of certain *G. morsitans* sub-species, where one crossing direction was less compatible than the reciprocal one (Curtis, 1972), the basis of such mating incompatibilities has been attributed to maternally inherited cytoplasmic factors (Gooding, 1987). Hence, with the discovery of *Wolbachia* in tsetse fly ovaries (O’Neill et al., 1993), the authors speculated that this well-known reproductive parasite might act as the causative agent for triggering post-mating isolation between tsetse fly species in nature. However, for acting as a true “speciation factor”, high prevalence and transmission frequency of maternally-transmitted *Wolbachia* are indispensable in order to avoid gene flow between emerging species (Coyne and Orr, 2004). Therefore it is of pivotal interest to thoroughly assign native *Wolbachia* infection frequencies throughout the genus *Glossina* from the field as well as from lab colonies.

**Fig. 1:**
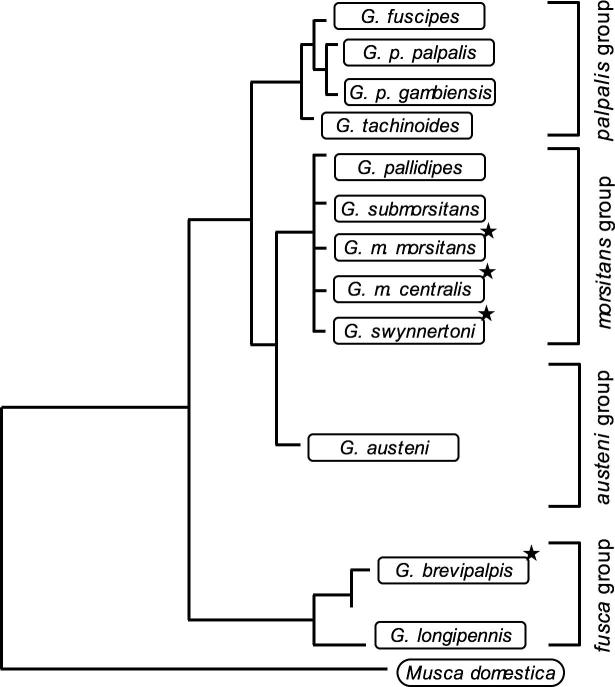
*Glossina* phylogeny. The phylogenetic tree of the genus *Glossina* is based on IST-2 sequence data and was adapted from Chen et al. (1999). The figure depicts four groups of *Glossina* spp.: *palpalis*, *morsitans*, *austeni*, and *fusca*. Tsetse flies from the *morsitans* (*G. m. morsitans*, *G. m. centralis*, *G. swynnertoni*) and the *fusca* group (*G. brevipalpis*) were analyzed in this study (indicated by asterisks).

Two recent studies have demonstrated the patchy distribution of *Wolbachia* in *Glossina* spp. Cheng et al. (2000) showed that infections among *G. swynnertoni* from field samples exhibited 11% *Wolbachia*-infection rate (Cheng et al., 2000), whereas *G. austeni* range from 0% to 98% and from 0% to 30% in *G. brevipalpis*. The most recent study on *Wolbachia-*prevalence within the genus *Glossina* came up with infection rates ranging from 0% to 100% in the *morsitans* group, from 0% to 8% in the *palpalis* group, and from 2% to 40% within the *fusca* group (Doudoumis et al., 2012; see Fig. 1 for *Glossina* phylogeny). Regarding the patchy *Wolbachia*-distribution demonstrated in both field and laboratory sampling sets of *Glossina* spp. (Cheng et al., 2000, Doudoumis et al., 2012), the question arises, whether these findings might be influenced by the inefficiency of standard molecular screening tools that do not detect *Wolbachia* low-titer infections. Indeed, detection of low-titer infections and reliable strain typing of closely related *Wolbachia* symbionts in insects has proven challenging and is dependent on the choice and information value of marker genes under consideration. Historically, the main body of *Wolbachia* strain-typing approaches and phylogenies were elaborated on the basis of sequence data derived from the 644-bp sequence of the highly dynamic *Wolbachia* Surface Protein gene *wsp* (Zhou et al., 1998), which is under strong adaptive evolution and a hotspot for inter-strain recombination (see Werren and Bartos, 2001). However, the *wsp* gene is not informative with respect to distinguish CI-inducing from neutral or even mutualistic strain phenotypes (Iturbe-Ormaetxe et al., 2005). Recently, *Wolbachia*-specific Multi Locus Strain Typing marker systems (MLSTs) were successfully used for phylogenetic strain typing (Baldo et al., 2006, Paraskevopoulos et al., 2006). For higher resolution of strain phylogenies and to distinguish even very closely related *Wolbachia* strains in different host systems we have recently developed new sets of hyper-variable marker systems covering mobile insertion sequences (IS elements) and Variable-Number-Tandem-Repeat (VNTR) loci (Riegler et al., 2005, Riegler et al., 2012, Miller and Riegler, 2006). As shown earlier, multiple-locus VNTR analysis (MLVA) is a highly successful method for studying genetic variability of many bacterial species, that was originally introduced for molecular typing of pathogens like *Bacillus anthracis* (Keim et al., 2000, Klevytska et al., 2001, Liao et al., 2006; and reviewed in Top et al., 2004, Lindstedt, 2005). Indeed, VNTRs have been proven to provide a high level of discriminatory power for strain differentiation because of their high susceptibility to mutation by replication slippage and ectopic recombination between cluster units (van Belkum et al., 1998). Based on the complete genome sequence of *Wolbachia* from *D. melanogaster w*Mel (accession number NC_002978.6; Wu et al., 2004), a novel set of hyper-polymorphic markers became available (Riegler et al., 2005), allowing for differentiation of *Wolbachia*-infections that share 100% identity at the *wsp* locus. Because the *wsp* locus provides low diagnostic resolution of the infection in different *Glossina* host species (Cheng et al., 2000, Doudoumis et al., 2012) we have adapted VNTR-fingerprinting to tsetse fly *Wolbachia*. Furthermore, for the detection of extreme low-titer infections that can easily escape standard *Wolbachia*-PCR methods, we have developed and successfully applied a novel *wsp*-PCR-blot technique (Arthofer et al., 2009, Miller et al., 2010).

By exploiting the improved arsenal of *Wolbachia*-specific marker loci at hand, in this study we have developed and applied novel, hypersensitive detection methods on the tsetse fly host system and found, (*i*) that the VNTR-141 locus is highly suitable for differentiating between *G. m. morsitans*, *G. m. centralis*, and *G. brevipalpis Wolbachia*-infections; (*ii*) the existence of multiple infections, *i.e.*, free cytoplasmic *Wolbachia*, and nuclear insertions of *Wolbachia* VNTR loci and IS elements into the host chromosome in some species of tsetse. Furthermore we demonstrate that (*iii*) in some *Glossina* species *Wolbachia*-infections can escape standard PCR detection methods by ‘hiding’ as low-titer infections, and that (*iv*) in *Glossina* hybrids maternally inherited *Wolbachia* easily escape symbiont titer regulation. Hence, similar to the situation found in some *Drosophila paulistorum* inter-semispecies hybrids (Miller et al., 2010), *Wolbachia* of *Glossina* spp. over-replicate intensively in hybrids. We speculate that disturbance of the native host–symbiont equilibrium in hybrids with mixed genetic host backgrounds can transform *Wolbachia* into pathogens by loss of replication control. This might consequently trigger hybrid incompatibilities between tsetse flies. Our study aims to highlight intimate *Wolbachia*-tsetse fly interactions in terms of symbiont fingerprinting, prevalence, and titer dynamics in four different *Glossina* species from laboratory and African field populations.

## 2 Materials and methods

### 2.1 Biological system

Individuals from both field populations and lab colonies deriving from the *morsitans* group of tsetse (*G. m. morsitans*, *G. m. centralis G. swynnertoni*), and the *fusca* group (*G. brevipalpis*) were screened for *Wolbachia*-infection via *Wolbachia*-specific PCR assays, and our PCR-blot technique plus sequence data analyses (see Table 1 for summary of analyzed samples).

**Table 1:** *Glossina* samples used for analyses sorted by species groups.

Species	Abbreviation	Group	wt/apo^a^	*n* Samples^b^	Origin
*G. morsitans morsitans*	*Gmm*	*morsitans* group	wt	2	Aksoy Lab, Yale School of Public Health, New Haven, USA
*G. morsitans morsitans*	*Gmm*	*morsitans* group	apo	1	Aksoy Lab, Yale School of Public Health, New Haven, USA
*G. morsitans morsitans*	*Gmm*	*morsitans* group	wt	5	Field collection from Republic of Zambia, Southern Africa
*G. morsitans morsitans*	*Gmm*	*morsitans* group	wt	35	Takáč Lab, Slovak Academy of Sciences, Bratislava, Slovakia
*G. morsitans centralis*	*Gmc*	*morsitans* group	wt	58	Insect Pest Control Laboratory FAO/IAEA, Vienna, Austria
*Gmm* × *Gmc* hybrids	*Gmm* × *Gmc*	*morsitans* group	wt hyb	68	Insect Pest Control Laboratory FAO/IAEA, Vienna, Austria
*G. swynnertoni*	*Gsw*	*morsitans* group	wt	10	Insect Pest Control Laboratory FAO/IAEA, Vienna, Austria
*Gsw* × *Gmc* hybrids	*Gsw* × *Gmc*	*morsitans* group	wt hyb	2	Insect Pest Control Laboratory FAO/IAEA, Vienna, Austria
*Gmc* × *Gsw* hybrids	*Gmc* × *Gsw*	*morsitans* group	wt hyb	3	Insect Pest Control Laboratory FAO/IAEA, Vienna, Austria
*Gsw* × *Gmm* hybrids	*Gsw* × *Gmm*	*morsitans* group	wt hyb	1	Insect Pest Control Laboratory FAO/IAEA, Vienna, Austria
*Gmm* × *Gsw* hybrids	*Gmm* × *Gsw*	*morsitans* group	wt hyb	2	Insect Pest Control Laboratory FAO/IAEA, Vienna, Austria
*G. brevipalpis*	*Gbr*	*fusca* group	wt	13	Insect Pest Control Laboratory FAO/IAEA, Vienna, Austria

a Samples are either wild-type (wt) or aposymbiotic (apo), wt hyb = wild-type hybrid.

b Number of independent individuals tested.

Antibiotic-treated, aposymbiotic *G. m. morsitans* (apo) were kindly provided from the Aksoy lab at Yale University, School of Public Health, USA. For generating these apo flies, wild type *G. m. morsitans* were maintained on blood meals supplemented with 1% (w/v) yeast extract (Becton Dickinson) and 20 μg/ml of tetracycline (B. Weiss, pers. comm.). Progeny of the treated parents are devoid of their endosymbionts, *Wigglesworthia*, *Sodalis*, and *Wolbachia.* Detailed treatment conditions can be found in Alam et al. (2011).

### 2.2 Generating *Glossina* hybrids

Hybrids were generated by setting up crosses between the following *Glossina* species (throughout the article females are named first in each cross): *G. m. morsitans* × *G. swynnertoni* (*Gmm* × *Gsw*), and *G. swynnertoni* × *G. m. morsitans* (*Gsw* × *Gmm*); *G. m. centralis* × *G. swynnertoni* (*Gmc* × *Gsw*), and *G. swynnertoni* × *G. m. centralis* (*Gsw* × *Gmc*) and *G. m. morsitans* × *G. m. centralis* (*Gmm* × *Gmc*). No hybrid offspring was produced in the reciprocal cross (*Gmc* × *Gmm*) (see also Table 1 for sample summary).

### 2.3 DNA extraction, VNTR, *wsp*, ISNew amplification, and cloning

Genomic DNA was extracted from whole bodies of adult *Glossina* females and males using the Puregene Gentra system and the QIAamp DNA Mini Kit (QIAGEN, Hilden, Germany). Additional DNA extracts from *G. m. morsitans* individuals were kindly provided by the Aksoy Lab from Yale School of Public Health, USA.

For diagnostic VNTR-141-PCR, protocols previously reported by Miller et al. (2010) were followed, using the primer set described by Riegler et al. (2005). Diagnostic *wsp*-PCRs for non-quantitative estimation of *Wolbachia* titer levels were performed according to Miller et al. (2010), using the primer set described by Jeyaprakash and Hoy, 2000. *Wolbachia* titer levels were also assessed via ISNew-PCR. Reactions were performed using one primer targeting the terminal inverted repeat sequence of the insertion sequence element ISNew (Wu et al., 2004). PCRs were performed in 10 μl reactions containing nuclease-free water, 1x reaction buffer, 3 mM MgCl_2_, 0.4 μM primer, 35 μM dNTPs, 0.04 U *taq* polymerase and 1 μl template. Thermal profile consisted of 2 min initial denaturation at 94 °C, followed by 35 cycles of 30 s at 94 °C, 45 s at 60 °C, and 1 min at 72 °C. Final extension was performed for 10 min at 72 °C. For all PCR reactions a Biometra T3000 Thermocycler (Biometra, Goettingen, Germany) was used. PCR products were purified using the peqGOLD Gel Extraction Kit (peqLab, Erlangen, Germany), inserted into the pTZ57R/T vector (Fermentas, St. Leon-Rot, Germany), and used to transform competent DH5α *Escherichia coli* cells. Clones containing the insert were cycle-sequenced with BigDye Terminator v3.1 at the Department of Marine Biology, University of Vienna, Austria. Sequences were analyzed using the BLAST algorithm, *G. morsitans* BLAST from the Sanger Institute GeneDB, and Geneious software (Biomatters Ltd.). All VNTR-141 sequences were deposited at GenBank under the accession numbers: JQ396432-JQ396437.

### 2.4 Quantitative Realtime-PCR

*Wolbachia*-titer levels in tsetse hybrids and corresponding parents were determined via quantitative Realtime-PCR (qRT-PCR). Primers amplifying a 77-bp fragment from *Wolbachia* 16S rRNA gene were designed (16SW_RTf 5′-CCTGATCCAGCCATGCCGCAT; 16SW_RTr 5-CGGCTGCTGGCACGGAGTTA and *Glossina tubulin* (Caljon et al., 2009) were used for normalization in all runs. Reactions containing 1x KAPA SYBR® FAST QPCR MasterMix (peqLab, Erlangen, Germany), nuclease-free water, 200 nM of each primer, and template at variable concentrations were run on a Mx3000Pro Stratagene Cycler using the following thermal profile: enzyme activation at 95 °C for 3 min followed by 45 cycles consisting of 3 s denaturation at 95 °C, 20 s primer annealing at 60 °C, and 6 s extension at 72 °C. Dissociation (melting curve) analysis was performed according to the instrument instructions.

### 2.5 PCR-blot technique

The PCR-blot technique, a combination of non-quantitative PCR and hybridization, allows for tracing even low titer *Wolbachia* by significantly enhancing the detection threshold. In the first part of the assay, standard *wsp*-PCR (see Section 2.3) followed by classic gel electrophoresis was performed on *Glossina* spp. samples. In the second part of the experiment, fragments were transferred onto a positively-charged nylon membrane via vacuum-blotting and hybridized overnight with a digoxigenin-labeled internal *wsp*-probe generated by PCR labeling. Chromogenic detection was performed using an alkaline phosphatase-conjugated anti-digoxigenin antibody and an NBT/BCIP combination. A detailed protocol is available in Arthofer et al. (2009).

### 2.6 Statistical testing

Significance in *Wolbachia*-infection titer shifts was estimated by calculating *P* values from unpaired *t*-tests using GraphPad Software (www.graphpad.com). Statistically significant differences were assumed when *P *< 0.05 (^*^), very significant when *P *< 0.01 (^**^), and extremely significant when *P *< 0.001 (^***^).

## 3 Results

### 3.1 Deciphering *Wolbachia* strain diversity and symbiont evolution in tsetse fly hosts

**Fig. 2:**
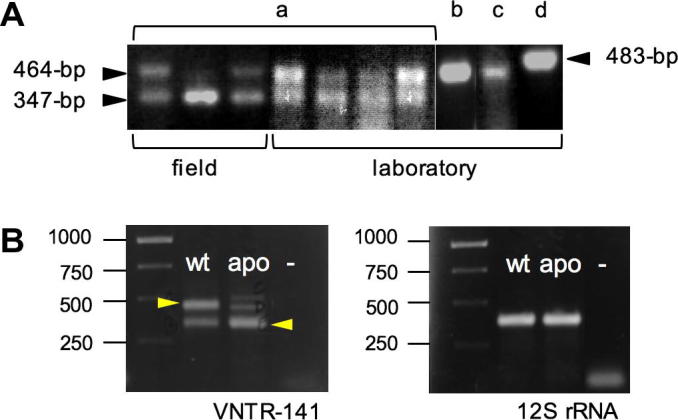
VNTR-fingerprinting of *Glossina* spp. (A) VNTR-141-PCR of four *Glossina* species. Samples are either laboratory-reared or field collected single individuals of (a) *G. m. morsitan Gmm*, (b) *G. m. centralis Gmc*, (c) *G. swynnertoni Gsw*, and (d) *G. brevipalpis Gbr.* Black arrowheads indicate a 347-bp fragment in *Gmm* of chromosomal origin; a 464-bp fragment of cytoplasmic origin in *Gmm*, *Gmc*, and *Gsw*; and a larger 483-bp fragment in *Gbr*. (B) VNTR-141-PCR of wild type (wt) and aposymbiotic (apo) *Gmm.* The cytoplasmic 464-bp band is more prominent in wt, whereas the nuclear 347-bp band is strong in the apo *Gmm*. DNA quality was assessed via 12S rRNA-specific PCR.

To assess full *Wolbachia*-diversity in tsetse flies from laboratory strains and field collections, we have applied a robust and highly sensitive VNTR-based screen on three members of the *morsitans* group, *i.e*., *G. m. morsitans*, *G. m. centralis* and *G. swynnertoni*, as well as on *G. brevipalpis* belonging to the distantly related *fusca* group (Fig. 1). VNTR-141 PCR on laboratory and field samples of *G. m. morsitans* results in two fragments of diagnostic length (347-bp and 464-bp, respectively; see Fig. 2A, lane a), whereas *Wolbachia* from *G. m. centralis* display only one amplicon of 464-bp (lane b). In *Wolbachia* from *G. swynnertoni*, the VNTR primer set amplifies a fragment similar in size to *G. m. centralis Wolbachia* (464-bp), but of weaker intensity (lane c). Finally the *G. brevipalpis* infection is characterized by a VNTR-141 fragment of unique size of 483-bp (lane d). The finding of two diagnostic VNTR-141 bands in all *G. m. morsitans* samples suggests either the existence of a double infection of the endosymbiont in *G. m. morsitans*, a duplication of the VNTR locus on the *G. m. morsitans Wolbachia* chromosome, or the horizontal transfer of the locus onto the host chromosome. Nuclear translocation events of *Wolbachia* genes have been reported in a variety of insect and nematode hosts (reviewed in Blaxter (2007)). Indeed, Doudoumis et al. (2012) reported recently on nuclear copies of at least three *Wolbachia* genes (16S rRNA, *wsp* and *fbpA*) in *G. m. morsitans* chromosomes. We determined the origin of the two VNTR-141 fragments via diagnostic PCR on wild type (wt) and apo (aposymbiotic; DNA kindly provided by S. Aksoy) *G. m. morsitans* samples. As shown in Fig. 2B, intensity of the upper VNTR band in wt flies is significantly decreased upon antibiotic treatment, suggesting a cytoplasmic origin, and the persistence of the smaller fragment in apo samples points towards a nuclear localization.

**Fig. 3:**
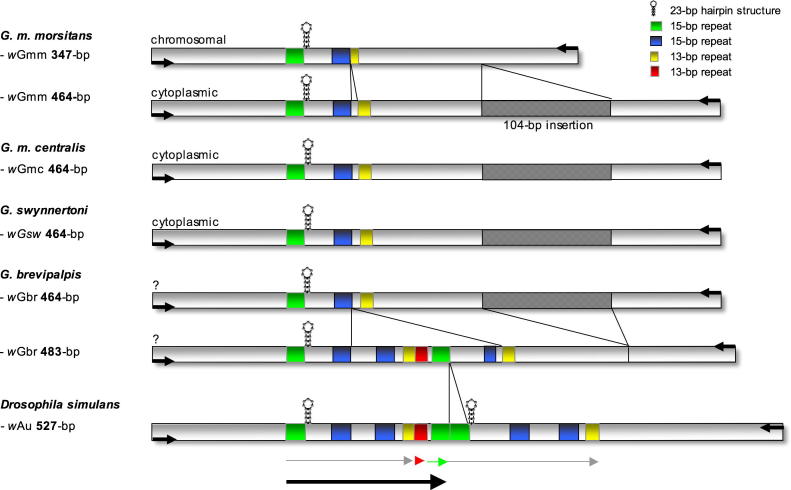
Organization of the VNTR-141 locus in *Glossina* spp. The 464-bp fragments of *wGmm*, *wGmc*, *wGsw*, and *wGbr* share a small 8-bp insertion between the 15-bp (blue) and 13-bp (yellow) repeat box and a 104-bp insertion (grid) at the 3′-end of the locus; the 347-bp chromosomal fragment of *Gmm* and the 483-bp *w*Gbr fragment lack both. Green and blue boxes represent 15-bp repeats, yellow and red ones 13-bp repeats. The gray arrow represents the complete core 108-bp repeat of *w*Au from *D. simulans*. Abbreviations: *w*Gmm, *w*Gmc, *w*Gbr, and *w*Gsw symbolize *Wolbachia* from *G. m. morsitans*, *G. m. centralis*, *G. brevipalpis*, and *G. swynnertoni*, respectively. Black arrow indicates the 141-bp master unit from *w*Mel-like strains.

In order to reconstruct the organization of the VNTR-141 locus in *Wolbachia* from *G. m. morsitans*, *G. m. centralis*, *G. brevipalpis*, and *G. swynnertoni*, PCR-amplified fragments were cloned and sequenced from multiple, independent tsetse fly candidates. Complete VNTR-141 repeat structure and composition have been recently described in detail in 10 *w*Mel-like *Wolbachia* strains of *Drosophila* spp. and the cherry fruit fly *Rhagoletis cerasi* (Riegler et al., 2012). According to the structural analyses performed by Riegler et al. (2012), we have aligned orthologous VNTR loci from *Glossina* spp. to *w*Au of *D. simulans*, harboring one complete 141-bp repeat unit (shown as a black arrow in Fig. 3). The 347-bp chromosomal fragment of *G. m. morsitans Wolbachia* represents the shortest and presumably most basic VNTR-141 locus structure described so far, consisting of an incomplete 108-bp core repeat which is characteristic of *w*Mel-like *Wolbachia* (shown as a gray arrow in *w*Au) by lacking the second 15-bp repeat B (blue box). In contrast, the VNTR-fragments amplified from *G. m. morsitans*, *G. m. centralis*, and *G. swynnertoni Wolbachia* are identical in size (464-bp) and sequence (>99%), including a small diagnostic 8-bp insertion between the two 15-bp repeat units (shown as blue and yellow boxes in Fig. 3). In addition cytoplasmic *Wolbachia* show a diagnostic *de novo* 104-bp insertion in the 3′ section of the VNTR locus of *G. m. morsitans*, *G. m. centralis*, and *G. swynnertoni Wolbachia* that is highly homologous to a disperse multi-copy insertion sequence found in the genome of *Culex pipiens Wolbachia w*Pip (at least two copies; accession number AM999887.1; Klasson et al., 2008). The absence of the 104-bp insertion in the orthologous VNTR loci of *Drosophila* spp. as well as *R. cerasi* implies a recent insertion event in the common ancestor of *morsitans* group *Wolbachia*. This assumption is corroborated by the fact that the 483-bp locus of *G. brevipalpis Wolbachia*, belonging to the distantly related fusca group is devoid of the 104-bp insertion but harbors both the full 108-bp core repeat period (gray arrow), and one complete 141-bp master unit (black arrow), similar to *w*Mel-like strains such as *w*Au *Wolbachia* of *D. simulans.* Interestingly, cloning and sequencing of the VNTR fragments from *G. brevipalpis* revealed an additional VNTR-141 locus of 464-bp size with high similarity in length, structure and sequence (>99%) to the cytoplasmic *Wolbachia* isolated from the *morsitans* group species (Fig. 3). Due to the slight size difference of 19-bp this second fragment was only detected by sequence analysis and might have been overlooked by applying a PCR-screen only. Hence, we suggest that *G. brevipalpis* harbors either two different cytoplasmic *Wolbachia* strains, or, similar to *G. m. morsitans*, also at least one translocated nuclear copy as a remnant of a former *Wolbachia* infection cycle (see discussion).

### 3.2 High fixation prevalence of *Wolbachia* in *G. morsitans* group species

**Fig. 4:**
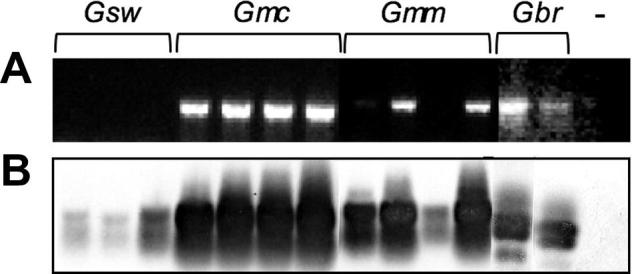
Detection of low-titer *Wolbachia* in tsetse flies. (A) *Wolbachia*-specific *wsp*-PCR of *Glossina* spp. (B) same gel after PCR-blot technique probed with a digoxygenin-labeled internal *wsp*-fragment (Arthofer et al., 2009). According to *wsp*-PCR, *Wolbachia*-infections are high in *G. m. centralis* (*Gmc*), intermediate in *G. m. morsitans* (*Gmm*), low in *G. brevipalpis* (*Gbr*), and not detectable in *G. swynnertoni* (*Gsw*). PCR-blot technique significantly enhances detection sensitivity, especially in *G. m. morsitans*, *G. brevipalpis* and *G. swynnertoni*. The *Wolbachia*-uninfected *D. simulans*, *STC* strain was used as negative control in both experiments.

According to *wsp-*PCR without combined blot-hybridization, *Wolbachia*-infections among *G*. *m. centralis*, *G. m. morsitans*, *G. swynnertoni*, and *G. brevipalpis* fall into four categories: high, intermediate, low, and not detectable (Fig. 4A). Based on this technique *G. m. centralis* infection is regarded as a high-titer infection, *G. m. morsitans* as intermediate, and *G. brevipalpis* as a low-titer infection. No *Wolbachia* was detected in *G. swynnertoni* samples via standard *wsp*-PCR. By applying our PCR-blot technique to the same sample set however, we significantly enhanced the detection capacity of our method and more samples tested positive for *Wolbachia*-infection. In *G. swynnertoni* 3/3 instead of 0/3 individuals and in 4/4 *G. m. morsitans* individuals were positive instead of only 2/4 (Fig. 4B). Via employing our highly sensitive detection tools, *i.e.*, PCR-blot technique and VNTR-based infection screen, to more samples from each tsetse species (data not shown) we estimated *Wolbachia-*prevalence as follows: *G. m. centralis* 57/58 (98%), *G. m. morsitans* 33/40 (83%); *G. swynnertoni* 7/10 (70%), and *G. brevipalpis* 9/13 (70%) respectively. These data suggest a generally high fixation rate of 70% on average in all tested *Glossina* species.

### 3.3 *Wolbachia* titer dynamics in *Glossina* hybrids

**Fig. 5:**
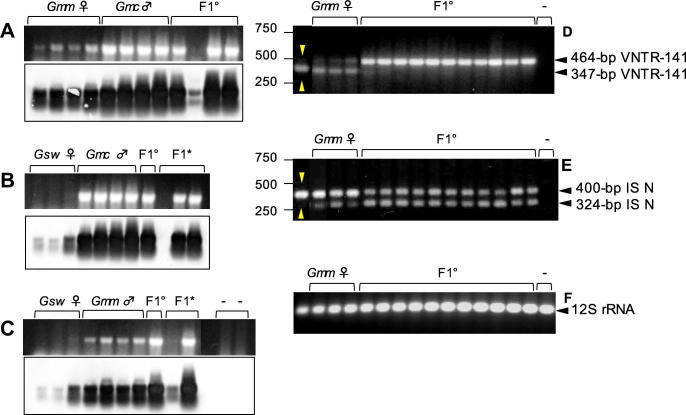
*Wolbachia*-prevalence in *Glossina* hybrids. (A–C) PCR-blot technique applied to parental and hybrid tsetse flies. Upper lanes show *wsp*-PCR, lower lanes show blot hybridizations using a digoxygenin-labeled internal *wsp*-probe (Arthofer et al., 2009). (A) *Gmm* mothers, *Gmc* fathers plus *Gmm* × *Gmc* F1 hybrids. (B) *Gsw* mothers, *Gmc* fathers, plus *Gsw* × *Gmc* and reciprocal hybrids. (C) *Gsw* mothers, *Gmm* fathers plus *Gsw* × *Gmm* and reciprocal hybrids. ♀ Symbolizes mothers, ♂ symbolizes fathers; hybrids are marked as F1° and F1^*^, respectively. F1° hybrids result from ♀ × ♂ cross, whereas F1^*^ hybrids result from the reciprocal cross. (D) VNTR-141-PCR on *Gmm* × *Gmc* F1 hybrids of both sexes showing 464-bp (cytoplasmic) and 347-bp chromosomal fragments. (E) ISNew-PCR on same sample set showing a 324-bp (cytoplasmic) and an *in silico* calculated 400-bp fragment (chromosomal). (F) Quality of DNA was assessed via 12S rRNA PCR. The *Wolbachia*-uninfected *Drosophila simulans* STC strain was used as negative control in all experiments.

Recent, independent studies reported on the importance of symbiont replication control, host background, and maintenance of a certain balance between host and symbiont loads (Poinsot et al., 1998, McGraw et al., 2002, Veneti et al., 2004, Bordenstein et al., 2006, Miller et al., 2010, Chafee et al., 2011, Login et al., 2011, Serbus et al., 2008, Serbus et al., 2011). In the *D. paulistorum* species complex, backgrounds from inter-semispecies hybrids negatively influence host fitness by boosting *Wolbachia*-titer (Miller et al., 2010). Furthermore a twofold titer increase of native *Wolbachia* was reported recently in the F1 hybrids between closely related parasitoid wasps of the genus *Nasonia* (Chafee et al., 2011). Here we generated hybrids between members of the *Glossina morsitans* group and assessed their *Wolbachia*-infection titer in comparison to the corresponding parental generation. By taking advantage of our PCR-blot technique, we revealed a dramatic increase of *Wolbachia*-titer in all hybrid backgrounds of *Gmm* × *Gmc* (the reciprocal cross does not produce living offspring); *Gsw* × *Gmc*, and *Gmc* × *Gsw*; *Gmm* × *Gsw*, and *Gsw* × *Gmm*, compared to their corresponding mothers (as *Wolbachia* is strictly maternally transmitted). As shown in Fig. 5, the vast majority of emerging hybrids exhibit significantly higher *Wolbachia*-titer levels than their mothers indicating over-replication of *Wolbachia* in mixed genomic backgrounds of tsetse flies (Fig. 5A–C; PCR in upper lanes; blot-hybridization in lower lanes). Crosses involving *G. swynnertoni* mothers show massive titer increase of *Wolbachia* in the F1 generation although these mothers harbor only low-titer infections (Fig. 5B and C). Applying two other primer sets to *Gmm* × *Gmc* hybrids targeting the VNTR-141 locus (Fig. 5D) and the *Wolbachia* insertion sequence element ISNew (Fig. 5E) generates similar results as for *wsp*-PCR. As expected for *G. m. morsitans* mothers, VNTR-141-PCR shows two clear bands that accord with the 347-bp chromosomal and the 464-bp cytoplasmic locus (Fig. 5D; and also see Fig. 2B). Whereas in apo *G. m. morsitans* samples the cytoplasmic 464-bp VNTR-band is no longer detectable (indicated by the arrow in Fig. 5D), all hybrids selectively amplify the cytoplasmic copy. Hence as the *Wolbachia*-titer levels are significantly enhanced in hybrid backgrounds, the cytoplasmic signal is much stronger and a primer bias towards the chromosomal target is circumvented. Increased titer in the F1 hybrids produces stronger cytoplasmic signals and this refers to the fact that only the higher band remains visible via gel electrophoresis. This massive titer increase of the symbiont in F1 *Gmm* × *Gmc* hybrids was also observed in PCR assays with the ISNew primer set, although in an opposite direction (Fig. 5E). Contrary to VNTR-141, where the longer 464-bp fragment selectively monitors symbiont titer dynamics in the cytoplasm, by means of ISNew PCR it is the smaller of the two fragments that is diagnostic for symbiotic *Wolbachia*. Here, apo samples selectively amplify the chromosomal 400-bp copies but lack the smaller 324-bp cytoplasmic fragment; whereas all hybrids show a clear intensity shift towards the cytoplasmic ISNew copies (324-bp) relative to *G. m. morsitans* mothers (Fig. 5E).

In order to determine *Wolbachia*-titer levels in parents and hybrids in a quantitative way, we performed *Wolbachia*-specific 16S rRNA qRT-PCR. Three-day old female and male F1 hybrids were analyzed for *Wolbachia*-infection level and compared to symbiont load in the corresponding 3-day old individuals from the parent colonies. Compared to PCR-hybridization analyses, qRT-PCR data support our finding of massive *Wolbachia*-titer increase in all five hybrid backgrounds.

**Fig. 6:**
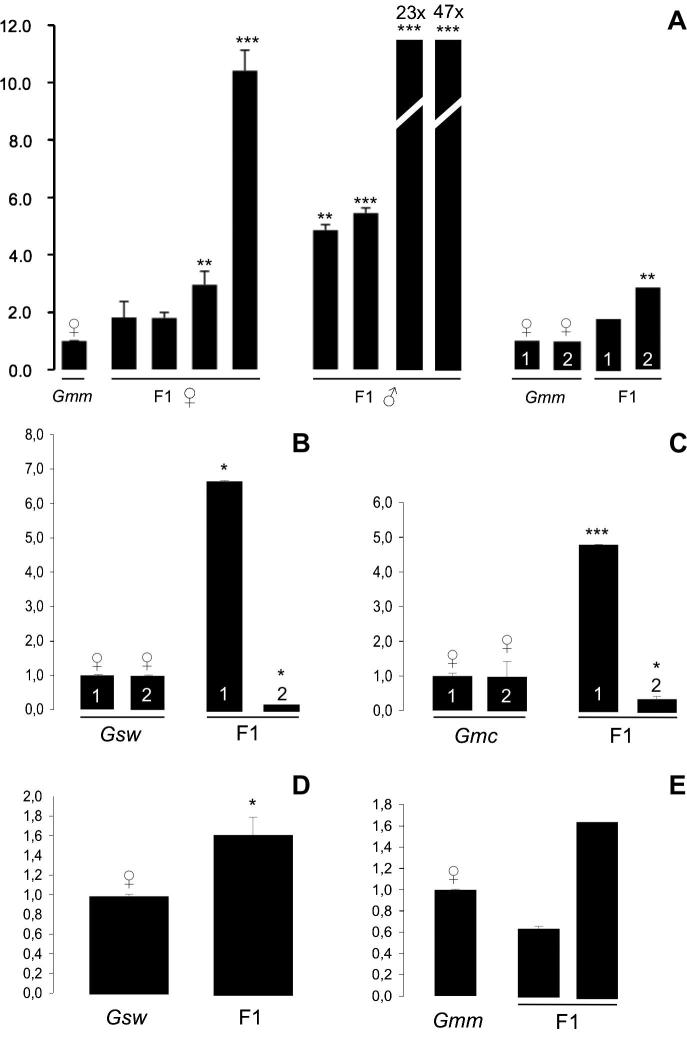
Quantitative 16S rRNA Realtime-PCR on *Glossina* parental females and respective hybrid offspring (females are named first). (A) *Gmm* × *Gmc* hybrid females (F1 ♀) and males (F1 ♂); ♀ symbolizes *Gmm* mothers; F1 indicates hybrid daughters and sons. (B) Offspring from *Gsw* × *Gmc* cross indicated by F1; ♀ symbolizes *Gsw* mothers. *Gsw* female one is the mother of F1 hybrid number one; *Gsw* female two is the mother of F1 hybrid two. Same applies to (C and E). In (D), only one hybrid with the corresponding mother is presented. (C) *Gmc* × *Gsw* hybrids symbolized by F1, *Gmc* mothers are marked by ♀, (D) F1 indicates a *Gsw* × *Gmm* hybrid and ♀ the *Gsw* mother, (E) *Gmm* × *Gsw* hybrids symbolized by F1; ♀ indicates the *Gmm* mother. Differences in symbiont titer levels were considered statistically significant when *P* < 0.05 (^*^), very significant when *P* < 0.01 (^**^), and extremely significant when *P* < 0.001 (^***^).

In *Gmm* × *Gmc* F1 generation, *Wolbachia*-titer is increased in 14 out of 15 hybrids when compared to corresponding *G. m. morsitans* mothers. In two tested hybrid males we measured extreme values of 23-fold and 47-fold increase of *Wolbachia*-titer. Overall, nine of these increased signals are supported by highly significant *P* values from statistical testing (see Fig. 6A and Supplement table for *P* values). Within the *Gmm* × *Gmc* F1 data set six hybrid daughters, seven hybrid sons, and three hybrids of unknown sex were included. Interestingly, sons generally show stronger *Wolbachia*-titer increase than daughters (mean 12-fold in sons compared to mothers *vs.* mean threefold in daughters compared to mothers; Supplementary table). Except for *Gmm* × *Gmc* crosses, the other four hybrid data sets are small which is due to the persistence of strong pre- and post-mating isolation mechanisms in these combinations (see discussion). Hence we had severe difficulties in obtaining enough emerging F1 offspring (Table 2). As shown in Fig. 6B *Wolbachia*-titer of *Gsw* × *Gmc* hybrids is increased significantly in one of the two tested F1 hybrids (sex not recorded). Here we observed a massive 6.7-fold increase of *Wolbachia*-level in hybrid 1 in comparison with the corresponding *G. swynnertoni* mother (*P *= 0.0187^*^), whereas in hybrid 2 the symbiont titer drops below the level of the corresponding mother (Fig. 6B and Supplementary table). Similar conflicting results on *Wolbachia* titer fluctuations were obtained from reciprocal crosses in the two tested F1 hybrids. Hybrid 1 is characterized by 4.8-fold increase (*P *= 0.0001^***^), whereas the symbiont titer in hybrid 2 is significantly decreased (0.4x; *P *= 0.0191^*^). Fig. 6D & E present *Wolbachia*-titer shifts determined in crosses involving *G. swynnertoni* and *G. m. morsitans* parents. *Gsw* × *Gmm* crosses almost fail to produce viable offspring (see discussion), which is reflected by only one F1 hybrid being available for *Wolbachia*-titer analyses. However, according to qRT-PCR, symbiont titer also increases in this hybrid background. We observed a 1.6-fold increase, which is considered statistically significant (*P *= 0.0421^*^). The reciprocal cross shows a significant *Wolbachia* titer increase in one out of two tested F1 hybrids. Although the 1.7-fold increase in infection titer is not statistically significant according to the *P* value calculated with an unpaired *t*-test (*P *= 0.1636) it still points towards a shift in *Wolbachia*-titer also in this hybrid background (see Fig. 6E). Importantly, similar to combinations between *G. swynnertoni* and *G. m. centralis* the sex of hatching F1 hybrids of *G. swynnertoni* and *G. m. moristans* parents was not recorded (see discussion).

**Table 2:** Hatching rates in *Glossina* inter-species hybrids.

	Cross (females first)	Cross set up	Pupae^a^	F1 hatch (numbers)^b^	F1 hatch (%)^c^
1	*Gsw* × *Gmm*	10	2	1	50
2	*Gmm* × *Gsw*	5	8	3	38
3	*Gsw* × *Gmc*	10	16	4	25
4	*Gmc* × *Gsw*	10	6	4	67
5	*Gmc* × *Gmm*	10	1	0	0
6	*Gmm* × *Gmc*	10	32	23	72
7	*Replicate of 6*	100	128	101	79

a Total numbers of pupae produced by the mating pairs for this cross breeding.

b Numbers of F1 hybrids hatched from the cross breeding.

c Percentage of F1 hybrids hatched from the cross breeding.

## 4 Discussion

### 4.1 *Wolbachia* strain diversity, prevalence and evolution in tsetse flies

As demonstrated earlier, VNTR-based *Wolbachia*-screens are informative tools for characterizing *Wolbachia-*infections in a diverse set of insect hosts such as *Drosophila* spp. and the cherry fruit fly *R. cerasi*, especially in cases where other symbiont marker systems like *wsp* and MLST are less informative (Riegler et al., 2005, Riegler et al., 2012, Miller and Riegler, 2006, Miller et al., 2010). Here, we show that VNTR-141 PCR is a robust and highly sensitive method to distinguish *Wolbachia*-infections also within the genus *Glossina*. *Wolbachia* from *G. brevipalpis*, belonging to the distantly related *fusca* group, can be differentiated unequivocally from the respective symbionts of *morsitans* group hosts, *i.e.*, *G. m. morsitans*, *G. m. centralis*, and *G. swynnertoni*, due to the presence of a higher molecular diagnostic band of the VNTR-amplicon (app. 480-bp). Cloning and sequencing of this band, however, elucidated the existence of two different VNTR loci of clearly distinctive *Wolbachia*-signatures and origin in *G. brevipalpis*. Although all three *Wolbachia*-infections from *G. m. morsitans*, *G. m. centralis*, and *G. swynnertoni* are characterized by VNTR-fragments of similar length (464-bp), the presence of a second, *G. m. morsitans*-specific *Wolbachia-*fragment (347-bp) provides a unique fingerprint profile for *G. m. morsitans*. By applying VNTR-PCRs to aposymbiotic *G. m. morsitans* flies (*Wolbachia* knock-down), as well as to *G. m. morsitans* × *G. m. centralis* hybrids (*Wolbachia* knock-in) we could rule out the potential existence of two different cytoplasmic *Wolbachia* strains in *G. m. morsitans* as well as a potential, but less likely, duplication of the VNTR-141 locus on a single *Wolbachia* chromosome. Hence we conclude that the smaller VNTR signals of 347-bp size stem from nuclear copies of a formerly translocated *Wolbachia* onto the *G. m. morsitans* host chromosome, whereas the bigger VNTR fragments of 464-bp length clearly derive from cytoplasmic copies of the endosymbiont. As shown recently by Doudoumis et al. (2012) *G. m. morsitans* chromosomes harbor fixed nuclear copies of the three *Wolbachia-*derived genes *wsp*, 16S *rRNA* and *fbpA.* Furthermore the authors have speculated that possibly bigger sections, or even the complete *Wolbachia* genome might have been translocated onto the *G. m. morsitans* chromosomes in recent evolutionary time after the split between *G. m. morsitans* and *G. m. centralis*. Hence the presence of a diagnostic chromosomal VNTR copy as well as ISNew copies in *G. m. morsitans* strongly support the presence of much larger translocated regions of the symbiont genome onto *G. m. morsitans* chromosomes (Doudoumis et al., 2012). Further expression studies on the potential activity of such symbiont-derived *de novo* nuclear genes in *G. m. morsitans* will be important to understand their functional and evolutionary implications. Finally, horizontal transfer (HT) events between *Wolbachia* and their natural hosts seem more common than earlier anticipated, since multiple independent cases have been reported recently in various insect as well as nematode host systems (Kondo et al., 2002, Fenn et al., 2006, Dunning Hotopp et al., 2007, Aikawa et al., 2009, Klasson et al., 2009, Nikoh et al., 2008, Nikoh and Nakabachi, 2009, Woolfit, 2009; and most recently Doudoumis et al., 2012).

Compared to the cytoplasmic 464-bp signal from *G. m. morsitans*, diagnostic VNTR fragments from *G. m. centralis* and *G. swynnertoni* differ in signal intensity. Whereas *Wolbachia* from *G. m. centralis* show a very intense VNTR amplicon, the signal is quite faint in *G. swynnertoni*, suggesting significant differences in respective titer levels. Complete absence of *wsp* signals, by means of standard PCR without blotting, in all tested *G. swynnertoni* samples confirms our assumption that *G. swynnertoni* is infected with low-titer *Wolbachia* that easily escape standard detection methods (see below).

To verify the reliability of our VNTR-141 primer set for *Wolbachia* fingerprinting in tsetse flies, we have cloned and sequenced VNTR-141 amplicons from all four *Glossina* species used in this study. Unexpectedly, in the eluted band of VNTR-PCR from *G. brevipalpis* we have detected two clearly distinctive VNTR variants, the diagnostic 483-bp copy, as well as a second but slightly smaller variant, almost identical to the 464-bp copies derived from *G. m. morsitans*, *G. m. centralis* and *G. swynnertoni*. The longer VNTR sequence is highly diagnostic and distinctive from the three *morsitans* group *Wolbachia* in terms of its locus organization, *i.e.*, repeat number and structure. Furthermore, this unique VNTR shows some structural similarities to the orthologous locus of *w*Au *Wolbachia* isolated from *D. simulans* (Miller and Riegler, 2006). This finding is not surprising as *G. brevipalpis* falls in another *Glossina* group (*fusca*) and is rather distantly related to the other three species, which are part of the *morsitans* group (Chen et al., 1999). Hence we conclude that *G. brevipalpis* is infected with a *w*Au-like *Wolbachia* found in various hosts of *Drosophila* spp. and *R. cerasi* populations harboring at least one complete unit of the 141-bp cluster repeat. This VNTR-type can be regarded as a quite ancestral stage of the diagnostic VNTR-141 locus which has expanded continuously in other *Wolbachia* hosts with up to seven copies in *w*Mel of *D. melanogaster* by means of slippage and/or unequal crossing (Riegler et al., 2012).

Besides this *w*Au-like *Wolbachia*, which is highly diagnostic for *G. brevipalpis* infection we have uncovered an additional VNTR sequence from *G. brevipalpis* stemming from a shorter VNTR-141 fragment that was obviously overlooked in gel electrophoresis. This can be explained by the marginal size difference existing between the two VNTR-141 fragments in *G. brevipalpis Wolbachia* (464- *vs.* 483-bp). A 19-bp size difference is too small to detect via classic PCR and gel electrophoresis and would need higher resolution gels or longer running conditions to separate the bands properly. The generation of apo *G. brevipalpis* lines will be necessary to uncover the evolutionary origin of the two VNTR loci. Similar to the situation in *G. m. morsitans*, we make the following assumptions: the additional VNTR fragment can either depict a hidden double *Wolbachia* infection, an intra-chromosomal duplication of the VNTR locus of the symbiont, or a horizontal transfer onto the *G. brevipalpis* chromosome*.* So far, we do not have experimental support allowing us to distinguish between any of the three assumptions on the origin of the two VNTR-types in *G. brevipalpis.*

However, the shorter VNTR from *G. brevipalpis* shares the unique 104-bp insertion and shows overall high sequence similarity (>99%) to the orthologous loci derived from *morsitans* group *Wolbachia*. Complete absence of complex repeat signatures (*i.e.*, lack of duplications of internal 15-bp repeats) in all four 464-bp VNTRs from *G. brevipalpis*, *G. m. morsitans*, *G. m. centralis* and *G. swynnertoni* implicates an evolutionary ancestral stage of this intergenic *Wolbachia* locus. Furthermore, this VNTR-type found in tsetse flies might represent the most basic VNTR locus type isolated so far, although the nuclear 347-bp copy of this locus on *G. m. morsitans* chromosomes lacks the diagnostic 104-bp insertion. Based on our BLAST search analyses the 104-bp insertion only matches with some as yet uncharacterized dispersed multi-copy repeats of *w*Pip *Wolbachia* from *Culex pipiens*, named here *IS*-Pip. Hence, we assume that this alien 104-bp section stems from a more recent *IS*-Pip insertion in the VNTR locus of cytoplasmic tsetse fly *Wolbachia*, presumably in the common ancestor of the *morsitans* group. However, complete absence of this diagnostic insertion in the chromosomal copy of the VNTR locus of *G. m. morsitans* suggests that this transfer into the nucleus took place before the 104-bp *IS*-Pip insertion event. In this case the nuclear copies can be regarded as remnants of an ancestral infection wave in *G. m. morsitans*. On the other hand, *G. m. centralis*, *G. swynnertoni* as well as *G. brevipalpis* are also infected with *Wolbachia* sharing the diagnostic *IS*-Pip insertion in the VNTR locus, suggesting that this *Wolbachia*-type was already present in their common host ancestor. Alternatively, different tsetse fly species, *i.e.*, members of the *fusca* as well as *morsitans* group, became recently and independently infected with a novel and highly parasitic *Wolbachia* strain from an outside source with the capacity of causing reproductive phenotypes such as CI, necessary for triggering their own propagation throughout host populations within short periods of time. Impressive short-time dynamics in global *Wolbachia* spreading throughout insect populations as well as species have been recorded from different natural insect hosts (Turelli and Hoffmann, 1995, Riegler and Stauffer, 2002, Riegler et al., 2005, Weeks et al., 2007). As recently shown, cytoplasmic *Wolbachia* of *G. m. morsitans* are capable of inducing strong unidirectional CI in crosses between apo females and naturally infected males (Alam et al., 2011). Here we demonstrate that *G. swynnertoni* is also infected with low-titer *Wolbachia* at high prevalence sharing identical VNTR signatures with cytoplasmic *G. m. morsitans* and *G. m. centralis Wolbachia* that over-replicate easily in hybrids (this study). We have earlier uncovered that even extreme low-titer *Wolbachia* infections, which also escape standard detection methods, have the capacity to express high bi-directional CI as well as complete male hybrid sterility in inter-semispecies hybrids of the *D. paulistorum* species complex (Miller et al., 2010).

Hence, we might speculate that tsetse fly *Wolbachia* of *G. swynnertoni*, *G. m. centralis* and perhaps at least one of the two strains detected in *G. brevipalpis* can be regarded as reproductive parasites with the capacity of inducing CI in their respective natural hosts. As demonstrated in our hybrid assays, maternally transmitted *Wolbachia* of *G. m. morsitans*, *G. m. centralis* and *G. swynnertoni* hosts massively over-replicate in the F1 hybrids. Future studies on titer levels in combination with quantitative fitness studies in apo *morsitans* and *fusca* group species, as well as in F1 hybrids between different *Glossina* species derived from apo parents are presently under way in our lab. Such studies will be essential to decipher the functional potential of tsetse fly *Wolbachia* in detail. Finally these results will have broader implication on the potential application of F1 hybrids of different tsetse fly species in fighting vector-borne diseases such as sleeping sickness and Nagana in Africa.

Besides their potential capacity of triggering reproductive phenotypes such as CI in a broader range of tsetse fly species and hybrids, naturally low titer *Wolbachia* can easily escape standard detection methods and hence affect estimates on their actual infection prevalence in the field as well as in lab colonies. Interestingly, in the most recent study assessing global *Wolbachia*-prevalence in tsetse flies Doudoumis et al. (2012) did not include *G. swynnertoni*. However, *Wolbachia*-infection in the species was analyzed in an earlier study (Cheng et al., 2000) that reported low prevalence of *Wolbachia* in African field populations of *G. swynnertoni* from Kenya (11%), but the authors did not include any laboratory-reared colonies. Here in contrast, we have screened only laboratory-reared *G. swynnertoni* individuals from FAO/IAEA Seibersdorf (Austria). As shown by Cheng et al. (2000) *Wolbachia*-prevalence in Kenyan field populations is quite low (11%). In the present screen, by applying our more sensitive PCR-blot technique, we have found only low-titer infections in *G. swynnertoni* that are, however, manifest at high prevalence (70%). If natural *Wolbachia*-titer in this species is below the standard detection threshold, assessing symbiont prevalence will be negatively biased and presumably explains the low prevalence in the Kenyan field populations reported earlier. Such technical limitations in standard detection methods can significantly affect estimates of natural symbiont load and frequency with severe consequences by missing the potential impact of low-titer *Wolbachia* strains on a diverse repertoire of host traits, such as host fitness, mating behavior and compatibilities, as well as insect immunity against pathogens like *Trypanosoma* and other human-relevant pests.

### 4.2 *Wolbachia* titer dynamics in *Glossina* hybrids and their potential role in host speciation

Mating incompatibilities between closely related tsetse fly species have been reported in earlier literature (Vanderplank, 1948; and reviewed in Gooding (1990)) and in the latter it was suggested that such maternally inherited incompatibilities might be explained by the presence of *Wolbachia* triggering high embryonic lethality and male sterility in hybrids (O’Neill et al., 1993). Furthermore, numerous studies have reported on the importance of symbiont titer maintenance and the consequences of its loss on the intimate host–symbiont interactions in a variety of insect and nematode host systems (*e.g.* Langworthy et al., 2000, McGraw et al., 2002, Bordenstein et al., 2006, Miller et al., 2010, Chafee et al., 2011, Serbus et al., 2011). In the species cluster *D. paulistorum*, hybrid backgrounds negatively influence host fitness by a massive increase of *Wolbachia*-infection (Miller et al., 2010). Another recent study demonstrated a twofold titer increase of native *Wolbachia* accompanied by proliferation into somatic host tissue in hybrids between closely related parasitoid wasps of the genus *Nasonia* (Chafee et al., 2011). In addition, Login et al. (2011) have recently shown that in a beetle host from the genus *Sitophilus* the load of its primary endosymbiont SPE is strictly regulated by the expression of the host antimicrobial peptide Coleoptericin-A (ColA). RNAi-knockdown of ColA causes loss of symbiont growth control resulting in disruption of the highly beneficial long-term relationship between host and symbiont (Login et al., 2011). Finally Serbus et al. (2011) have demonstrated that *Wolbachia*-titer in *D. melanogaster* might be regulated through a feedback loop between the host gene *gurken* (*grk*) and the symbiont itself. Regarding our finding that *Wolbachia*-titers increase dramatically in the five *Glossina* hybrids compared to their non-hybrid mothers, we suggest that strict titer regulation of the symbiont is crucial in that system too. Here we demonstrate statistically significant *Wolbachia*-titer increase in the majority of the tested *Glossina* F1 hybrids via qRT-PCR. These results were verified by two more independent molecular techniques, *i.e.*, combined PCR-blot technique and non-quantitative *Wolbachia*-specific VNTR-PCR.

#### 4.2.1 Hybridization of *G. swynnertoni* with *G. m. morsitans* and reciprocal cross

As earlier reported, *G. swynnertoni* males are able to both inseminate and fertilize *G. m. morsitans* females (Gooding, 1993, Gooding, 1997). In our crosses of *Gsw* × *Gmm* 10 mating pairs produced only two pupae (80% failure rate; Table 2), and only one of these emerged. This F1 hybrid exhibited a 1.6-fold increase of *Wolbachia*-titer in 16S rRNA qRT-PCR screens. As both parents are infected with two presumably slightly different *Wolbachia* strains we have probably observed a novel case of bidirectional CI, leading to extremely high rates of embryonic death at early onset (Yen and Barr, 1971, Daniels and Ehrman, 1974, Ehrman and Daniels, 1975). However, since tsetse flies are adenoviviparous and retain the egg and developing larva in the uterus until ready to pupate, we cannot distinguish between early or late hybrid mortality during their development. *Wolbachia*-triggered hybrid mortality has been described earlier for neotropical *D. paulistorum*, where, despite their mainly low-titer *Wolbachia*-infection, hybridization in certain inter-semispecies hybrids leads to high levels of early embryonic lethality plus complete hybrid male sterility (Miller et al., 2010). Our findings reported here suggest that something similar to *D. paulistorum* is exhibited in the *Gsw* × *Gmm* hybrids, where the maternal *Wolbachia* is a low-titer infection according to our PCR-blot screening technique. Alternatively, partial failure of the *G. swynnertoni* mothers to transfer living sperm to the spermatheca and keep it there post-insemination can explain the low numbers of F1 hybrid offspring (Gooding, 1993) but this was not tested here.

In reciprocal crosses, slightly more pupae were produced from even less mating pairs (five pairs produced eight pupae, three of which emerged; see Table 2), suggesting that post-mating barriers to hybridization are weaker. The described phylogenetic relationship of the two species reflects the hybridization behavior observed in *Gsw* × *Gmm*: *G. swynnertoni* is more closely related to *G. m. centralis* than to *G. m. morsitans* (reviewed in Gooding and Krafsur (2005)) and it was shown that only *G. m. centralis* males are highly successful in fertilizing *G. swynnertoni* females, whereas the more distant *G. m. morsitans* males are not (Gooding, 1997). We have determined a 1.7-fold *Wolbachia-*titer increase in *Gmm* × *Gsw* hybrids, which is almost the same as in the reciprocal cross (1.7-fold *vs.* 1.6-fold). The native *Wolbachia*-titers of *G. swynnertoni* and *G. m. morsitans* mothers differ significantly but we suggest that this is not important in the hybrids since titer up-regulation does not correspond to native maternal titer levels. Interestingly, the level of titer increase differs significantly between the two tested *Gmm* × *Gsw* hybrids in this study. We suggest that this might eventually be shown to correspond to the sex of the hybrids, pointing towards an enhanced tolerance of male hybrids to *Wolbachia*-titer increase. Similar results were obtained for *Gsw* × *Gmc* and the reciprocal cross (see below). We have demonstrated that F1 sons from *Gmm* × *Gmc* crosses exhibit a mean increase in *Wolbachia* titer, four times higher than the F1 daughters, a finding that supports our idea of hybrid males tolerating higher symbiont loads than hybrid females. In order to test this hypothesis, we will re-evaluate *Wolbachia*-titer levels in sexed F1 hybrids of *Gsw* × *Gmm* and the reciprocal cross.

#### 4.2.2 Hybridization of *G. swynnertoni* with *G. m. centralis* and reciprocal cross

In our study, 10 mating pairs between *G. swynnertoni* females and *G. m. centralis* males produced 16 pupae but only four of these emerged, which represents a quite low emergence rate of only 25% (Table 2). This rate, however, is not much lower than the one reported by Gooding, 1997 (30%). In qRT-PCR we monitored a massive increase in *Wolbachia*-titer in one out of the two tested F1 hybrids (6.7-fold). Natural hybrids between *G. m. centralis* and *G. swynnertoni* were observed previously (Lloyd, 1935, Potts, 1937, Vanderplank, 1947, Vanderplank, 1948) and this is not unlikely since *G. swynnertoni* and *G. m. centralis* are regarded as most closely related within the *morsitans* group (Gooding, 1997). No rejection of *G. m. centralis* males by *G. swynnertoni* females has been reported, and hence both *G. m. morsitans* and *G. m. centralis* males are able to inseminate and fertilize *G. swynnertoni* females successfully (average 30%; Gooding, 1997). This suggests that pre-mating barriers to hybrid formation are, if they exist, very weak. Post-mating barriers are obviously present, since in our crosses, 75% of pupae failed to develop further (Table 2), probably due to delayed lethality during pupation. We speculate that low emergence rates might be caused by over-replication of the symbiont in hybrid pupae, although this scenario needs further experimental proof by qRT-PCR on early and late pupal stages of parental and hybrid samples.

In the reciprocal cross (*Gmc* × *Gsw*), overall adult emergence was higher (67%) but fewer pupae were produced (also see Table 2). *G. m. centralis* females are more likely to reject *G. swynnertoni* males with *G. m. centralis* females rejecting more than half of the offered *G. swynnertoni* males (Gooding, 1993). General difficulties in mating *G. m. centralis* females to *G. swynnertoni* males have also been reported (Potts, 1937). *Wolbachia*-titer increase in *Gmc* × *Gsw* hybrids was lower than in the reciprocal cross (4.8-fold *vs*. 6.7-fold). As mentioned above, titer levels are significantly different between the two *Gmc* × *Gsw* hybrids, and this strong variability was also observed in the four hybrids of *Gsw* × *Gmc* and the reciprocal cross. In each set, one hybrid exhibited more than 10-fold greater titer increase than the other one, which might be due to a significantly higher tolerance of males to increased symbiont load. As already mentioned for hybrids resulting from *Gmm* × *Gsw* cross breeding, this hypothesis will be tested in follow-up experiments with newly generated sexed hybrids.

#### 4.2.3 Hybridization of *G. m. morsitans* with *G. m. centralis* and reciprocal cross

Previous studies have reported that *G. m. morsitans* females can be easily mated to *G. m. centralis* males (Curtis, 1972, Gooding, 1983) and no obvious barriers to mating or sperm transfer exist (Gooding and Jordan, 1986). However, all F1 hybrid males are sterile (reviewed in Gooding (1999)). These data are consistent with the hybridization success in our crossing experiment. High numbers of offspring, compared to the other cross combinations, were obtained from *Gmm* × *Gmc* crosses. Ten mating pairs produced 32 pupae, and 23 of these emerged (72% emergence rate). A second replicate with 100 mating pairs produced 128 pupae from which 101 emerged (79% emergence rate; Table 2). The increase of *Wolbachia*-titer in *Gmm* × *Gmc* hybrids exhibited high variability, ranging from 1.2-fold to 47.0-fold. Again we observed a significant difference between hybrid females and males: on average, titer levels in females were increased 3-fold but 12-fold in males. This finding suggests a potential higher tolerance of hybrid males to *Wolbachia*-titer increase than females. As mentioned earlier, the F1 hybrids resulting from the four other cross combinations with *G. swynnertoni* also exhibited strong variability in symbiont titer levels but, contrary to *Gmm* × *Gmc* hybrids, with significantly reduced symbiont titer levels in approximately half of the emerging hybrids. However, we need to test further the theory of imbalanced tolerance of male and female tsetse hybrids to symbiont load by generating novel hybrids and assessing their *Wolbachia*-titers in both sexes. Although earlier studies have shown that *G. m. morsitans* and *G. m. centralis* can hybridize in both directions, generally less F1 hybrids are obtained when *G. m. centralis* females were used (reviewed in Gooding (1999)). In our crossing experiment, 10 mating pairs failed to produce living F1 *Gmc* × *Gmm* offspring, which is inconsistent with the data available from the literature. In our assays 10 mating pairs produced only one pupa that did not emerge (0% emergence rate; Table 2), suggesting that either mating was mainly prevented or females were inseminated (transfer of sperm) but hybrid embryos did not develop properly. In the latter case, we might see, similar to the situation in *Gsw* × *Gmm*, extremely high levels of embryonic lethality triggered by bidirectional CI, similar to the situation in *Gsw* × *Gmm*. This is supported by a recent publication from Alam et al. (2011) that reports the existence of unidirectional CI-causing *Wolbachia* infection in matings between apo *Gmm* females and naturally infected *Gmm* males.

## 5 Conclusions

By the application of our improved *Wolbachia* detection tools, *i.e.*, VNTR-fingerprinting, blot PCR, and artificial titer enhancement in hybrids we have succeeded in uncovering low-titer infections in tsetse fly species and lab colonies that were earlier overlooked by standard *Wolbachia* detection methods. Furthermore, by means of our hypersensitive detection tools we have assigned much higher infection frequencies than earlier reported in tsetse flies from field populations and lab colonies. In addition to the power of this high-resolution method we have also demonstrated the capacity of tsetse fly *Wolbachia* to induce CI in hybrids between *Glossina* species that presumably triggers incipient speciation in tsetse flies. According to previous studies, barriers to hybrid formation in the genus *Glossina* are weak since natural hybridization between the species has been reported repeatedly (reviewed in Gooding (1990)). In this study, however, we have observed barriers to hybridization on both post- and pre-mating levels. Interestingly, Gooding has suggested in one of his recent publications (Gooding and Krafsur, 2005) that mating barriers are already in the process of formation and that tsetse flies could serve as a suitable system for studying ongoing speciation. As in the situation observed in hybrids between the semispecies of the *D. paulistorum* species cluster (Miller et al., 2010), *Glossina* hybrids are prone to male sterility and reduced fecundity of females (Gooding, 1993). Here, we show that *Wolbachia* titers are massively increased in most F1 hybrids and speculate that this over-replication could trigger the aforementioned strong post-mating isolation. We suggest that we probably uncovered the induction of *Wolbachia*-induced CI in cross breeding of *morsitans* group taxa, causing high levels of early embryonic and/or late pupal lethality. Regarding the finding that both parents harbor *Wolbachia* at high prevalence and that F1 hybrid formation is affected in both directions, not only uni- but also bidirectional CI in tsetse flies is quite likely. As earlier demonstrated in *Culex pipiens*, parasitoid wasps of the genus *Nasonia* and neotropical *Drosophila* species expression of strong bidirectional CI can be regarded as effective post-mating barriers capable of fostering incipient host speciation (Yen and Barr, 1971, Bordenstein et al., 2001, Miller et al., 2010).

## Disclosures

The authors Daniela I. Schneider, Kathrin I. Garschall, Andrew G. Parker, Adly M.M. Abd-Alla and Wolfgang J. Miller report no conflicts of interest to be declared.
