# Supplementary material for: Global Wolbachia prevalence, titer fluctuations and their potential of causing cytoplasmic incompatibilities in tsetse flies and hybrids of Glossina morsitans subgroup species
Source: J Invertebr Pathol. 2013 Mar;112(Suppl 1):S104–15. doi: 10.1016/j.jip.2012.03.024 (PMC3625123; doi:10.1016/j.jip.2012.03.024)
Supplement: Supplementary data 1 [file mmc1.doc]

| **Cross** | **x-fold titer** | **Standard deviation** | ***P*-value**^a^ | **Assay** |
| --- | --- | --- | --- | --- |
| *Gmm x Gmc* |  |  |  |  |
|  |  |  |  |  |
| F1 female hybrid | 1,4 | 0,01 | 0,4086 | *vs.* *Gmm* pooled mothers |
| F1 female hybrid | 1,6 | 0,10 | 0,1818 | *vs.* *Gmm* pooled mothers |
| F1 female hybrid | 1,8 | 0,05 | 0,1062 | *vs.* *Gmm* pooled mothers |
| F1 female hybrid | 1,8 | 0,55 | 0,1977 | *vs.* *Gmm* pooled mothers |
| F1 female hybrid | 2,9 | 0,19 | 0,0084 ** | *vs.* *Gmm* pooled mothers |
| F1 female hybrid | 10,4 | 0,47 | 0,0001 *** | *vs.* *Gmm* pooled mothers |
| mean increase daughters | 3x |  |  |  |
|  |  |  |  |  |
| F1 male hybrid | 1,2 | 0,85 | 0,6759 *NS* | *vs.* *Gmm* pooled mothers |
| F1 male hybrid | 2,1 | 0,16 | 0,0476 * | *vs.* *Gmm* pooled mothers |
| F1 male hybrid | 2,9 | 0,59 | 0,0227 * | *vs.* *Gmm* pooled mothers |
| F1 male hybrid | 4,9 | 0,71 | 0,0027 ** | *vs.* *Gmm* pooled mothers |
| F1 male hybrid | 5,4 | 0,19 | 0,0004 *** | *vs.* *Gmm* pooled mothers |
| F1 male hybrid | 22,9 | 3,13 | 0,0003 *** | *vs.* *Gmm* pooled mothers |
| F1 male hybrid | 47,0 | 2,07 | 0,0001 *** | *vs.* *Gmm* pooled mothers |
| mean increase sons | 12x |  |  |  |
| F1 hybrid # 1 | 1,8 | 0,15 | 0,0765 | *vs.* mother # 1 |
| F1 hybrid # 2 | 2,9 | 0,13 | 0,0094 ** | *vs.* mother # 2 |
| F1 hybrid # 3 | 0,9 | 0,37 | 0,4895 | *vs.* mother # 3 |
| *Gsw x Gmc* |  |  |  |  |
| F1 hybrid # 1 | 6,7 | 0,02 | 0,0187 * | *vs.* mother # 1 |
| F1 hybrid # 2 | 0,2 | 0,02 | 0,1804 | *vs.* mother # 2 |
| *Gmc x Gsw* |  |  |  |  |
| F1 hybrid # 1 | 4,8 | 0,01 | 0,0001 *** | *vs.* mother # 1 |
| F1 hybrid # 2 | 0,4 | 0,08 | 0,0191 * | *vs.* mother # 2 |
| *Gsw x Gmm* |  |  |  |  |
| F1 hybrid | 1,6 | 0,18 | 0,0421 * | *vs.* mother |
| *Gmm x Gsw* |  |  |  |  |
| F1 hybrid # 1 | 0,6 | 0,36 | 0,4710 | *vs.* Gmm mothers |
| F1 hybrid # 2 | 1,7 | 0,02 | 0,1636 | *vs.* Gmm mothers |

**Supplement Table.** *Wolbachia*-titers in parental females and corresponding *Glossina* inter-species hybrids. ^a^Two-tailed *P* values from unpaired t-tests; differences in symbiont titer levels were considered statistically significant when *P* > 0.05 (*); very significant when *P* > 0.001 (**), and extremely significant when *P* > 0.0001 (***).
